# Multilevel Analysis of the Food and Physical Activity Environment and Adult Obesity Across U.S. Counties and States

**DOI:** 10.3390/ijerph23020142

**Published:** 2026-01-23

**Authors:** Ann Mary Abraham, Michael D. Swartz, Alexandra E. van den Berg, Stephen H. Linder

**Affiliations:** 1 Department of Management, Policy and Community Health, School of Public Health, The University of Texas Health Science Center at Houston (UT Health), Houston, TX 77030, USA; abrahamann32@gmail.com; 2 Department of Biostatistics, School of Public Health, The University of Texas Health Science Center at Houston (UT Health), Houston, TX 77030, USA; michael.d.swartz@uth.tmc.edu; 3 Department of Health Promotion and Behavioral Sciences, School of Public Health, The University of Texas Health Science Center at Houston (UT Health), Austin Campus, Austin, TX 78701, USA; alexandra.e.vandenberg@uth.tmc.edu; 4 Michael & Susan Dell Center for Healthy Living, School of Public Health, The University of Texas Health Science Center at Houston (UT Health), Austin Campus, Austin, TX 78701, USA; 5 Institute for Health Policy, School of Public Health, The University of Texas Health Science Center at Houston (UT Health), Houston, TX 77030, USA

**Keywords:** adult obesity, food environment, physical activity environment, multilevel modeling, U.S. counties, socioeconomic disparities, SNAP benefits, food insecurity, recreational access, public health policy

## Abstract

**Highlights:**

**Public health relevance—How does this work relate to a public health issue?**

- Adult obesity remains a major public health challenge in the United States, with substantial geographic and social disparities across counties and states.
- This study examines how food access, physical activity environments, and socioeconomic conditions are associated with adult obesity at the county and state level.

**Public health significance—Why is this work of significance to public health?**

- The analysis uses a national multilevel framework to distinguish between county-level and state-level contextual influences on obesity prevalence.
- The findings highlight that structural and environmental conditions are linked with obesity patterns, even after accounting for socioeconomic and demographic factors.

**Public health implications—What are the key implications or messages for practitioners, policy makers and/or researchers in public health?**

- Results suggest that efforts to address obesity may benefit from considering local food environments, economic conditions, and access to physical activity resources together rather than in isolation.
- The findings underscore the importance of place-based and context-sensitive approaches when designing obesity prevention strategies.

**Abstract:**

Adult obesity rates have risen steadily across the United States over the past decade, with more than 40% of adults affected. Persistent geographic and demographic disparities exist in obesity prevalence across the nation. While prior research has examined individual or environmental associated factors of obesity, limited studies have addressed both physical activity and food environments across the nation using multilevel approaches. This cross-sectional ecological study (2014–2024) used a two-level random intercept model to assess the association between county- and state-level factors and adult obesity prevalence across over 3000 U.S. counties nested within 51 states. County-level associated factors included food insecurity, poverty, unemployment, median household income, limited access to stores, and the density of various food outlets (grocery stores, convenience stores, supercenters, fast-food restaurants, Supplemental Nutrition Assistance Program (SNAP)-authorized retailers, and farmers’ markets), along with access to recreational facilities. State-level factors included SNAP benefits per capita and the presence of soda and chip taxes. Variables were group-mean- or grand-mean-centered to distinguish within- and between-state effects. Results showed that food insecurity, poverty, unemployment, limited access to stores, and a higher density of fast-food and convenience stores were positively associated with adult obesity prevalence. While higher recreational facility access, supercenter availability, median household income, SNAP benefits per capita were associated with lower adult obesity prevalence, these associations varied in strength across counties and states. These results emphasize the need for place-based strategies that address both the physical activity and food environment in shaping obesity disparities.

## 1. Introduction

Adult obesity remains one of the most pressing public health challenges in the United States, with more than 40% of adults currently affected and prevalence continuing to rise across most regions [1,2]. The burden of obesity is unevenly distributed, with persistent geographic, socioeconomic, and racial disparities documented in both urban and rural contexts. A large body of research demonstrates that features of the food and physical activity (PA) environment—such as access to recreational spaces, density of food outlets, and local economic conditions—substantially shape obesity risk beyond individual lifestyle behaviors [3,4,5,6].

National studies have shown meaningful regional variation in food and PA environments. For example, substantial differences in park access, walkability, and built-environment indicators across U.S. counties have been documented, with strong associations to obesity and physical inactivity patterns [7,8]. Similarly, prior national or multi-state analyses have documented that counties in the South and Midwest tend to have higher concentrations of convenience stores and fast-food outlets, while the Northeast and West generally have more supermarkets, recreational resources, and lower obesity rates [5,6,9]. Yet, despite these contributions, many studies rely on limited geographic regions, focus on either food or PA environments—but not both—or do not incorporate a multilevel ecological perspective.

The environmental variables examined in this study are also well-established in the prior literature. Fast-food and convenience store density have consistently been linked to higher obesity risk, particularly in low-income or minority communities [10,11]. In contrast, greater supermarket access, farmers’ markets, and recreational facilities have been associated with healthier diets, more physical activity, and lower obesity prevalence [9,10,11,12,13]. Economic conditions—including food insecurity, poverty, unemployment, and median household income—strongly influence diet quality and the capacity to access healthy foods [10,14]. State-level policies, such as Supplemental Nutrition Assistance Program (SNAP) benefits and taxes on sugar-sweetened beverages or snack foods, further shape population-level dietary behaviors and purchasing power [15,16,17].

However, significant gaps remain. First, relatively few studies link both food and PA environments into a combined national multilevel framework using consistent county- and state-level measures. Second, prior work seldom examines how these environments operate differently across counties with varying racial and ethnic compositions, despite substantial evidence that structural inequities, segregation, and racialized economic disadvantage modify environmental exposures. Third, many national studies rely solely on descriptive ecological comparisons rather than multilevel modeling that can separate within-state from between-state effects.

This study addresses these gaps by conducting a national multilevel analysis across all U.S. counties and states, using harmonized data from County Health Rankings and the USDA Food Environment Atlas. The purpose of this study is to estimate the association between food environment characteristics, physical activity resources, socioeconomic conditions, and adult obesity, while accounting for state-level policy influences and demographic composition. By integrating county- and state-level predictors, this study provides a more comprehensive understanding of how local environments and broader structural contexts jointly shape adult obesity disparities across the United States.

## 2. Materials and Methods

### 2.1. Study Design and Data Sources

This is a retrospective ecological study of counties across all fifty U.S. states and the District of Columbia during the period 2014–2024, comprising approximately 3194 county-level observations. County-level estimates of adult obesity prevalence were obtained from the County Health Rankings, which in turn draws from the CDC’s PLACES project. PLACES uses Behavioral Risk Factor Surveillance System (BRFSS) data combined with model-based small area estimation techniques to generate local estimates [18]. Additional covariates, including food environment measures, were drawn from the USDA Food Environment Atlas and related data sources [19]. The study was deemed exempt from review by the Institutional Review Board (IRB) through Student Research at the University of Texas School of Public Health, Houston, as it utilized publicly available, de-identified data.

### 2.2. Study Hypotheses

This study examined whether county- and state-level environmental, socioeconomic, and demographic factors were associated with adult obesity prevalence. The hypotheses are specified below.

#### 2.2.1. Food Environment

- Counties with more grocery stores and farmers’ markets (including those selling fruits and vegetables) would have lower adult obesity prevalence.
- Counties with higher densities of fast-food restaurants, convenience stores, and supercenters, and with greater proportions of low-access households, would have higher adult obesity prevalence.

#### 2.2.2. Physical Activity Environment

- Counties with greater access to recreational and fitness facilities would have lower obesity prevalence.

#### 2.2.3. Socioeconomic Factors

- Higher median household income would be associated with lower obesity rates.
- Higher poverty, food insecurity, and unemployment would be associated with higher obesity rates.

#### 2.2.4. Racial/Ethnic Composition

- Counties with higher proportions of racial/ethnic minority populations were expected to show variation in obesity prevalence (direction varying by group based on the prior literature).

#### 2.2.5. State-Level Policy Context

- States with higher Supplemental Nutrition Assistance Program (SNAP) benefits per capita and higher food tax levels were expected to have lower adult obesity prevalence.

### 2.3. Theoretical Framework

This study is based on the Socio-Ecological Model (SEM), which emphasizes that health behaviors and outcomes are shaped by multiple factors at different levels, including individual, community, and policy environments. In the context of obesity, environmental and policy-level determinants like food outlet distribution, recreational resources, socioeconomic conditions, and state policy environments. These factors play an important role in shaping opportunities for healthy behaviors. The SEM therefore supports examining how local food and physical activity environments interact with wider structural contexts to influence adult obesity across U.S. counties.

### 2.4. Study Conceptual Framework

**Figure 1:**
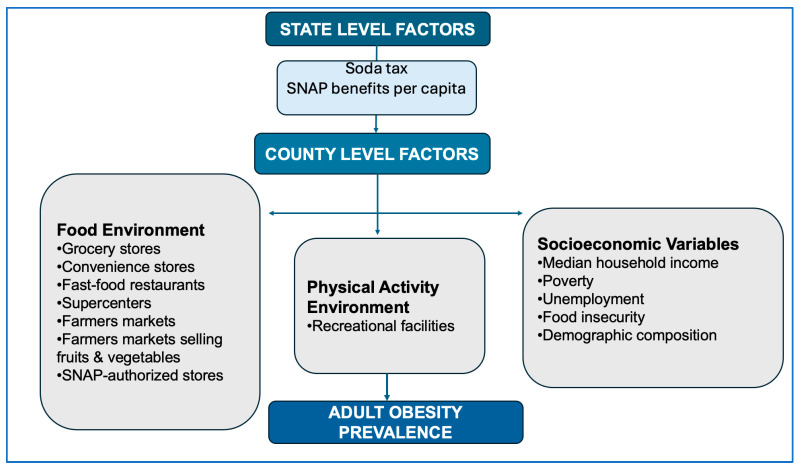
Conceptual framework illustrating state-level and county-level environmental, socioeconomic, and demographic factors influencing adult obesity.

Guided by the SEM, this study groups the key determinants of adult obesity into four categories: (1) food environment factors, (2) physical activity environment factors, (3) socioeconomic and demographic characteristics, and (4) state-level policy influences. These determinants align with the study’s hypotheses and the multilevel modeling analysis. Figure 1 (conceptual framework) summarizes these multilevel pathways, illustrating how county-level and state-level contextual factors are associated with variations in adult obesity prevalence.

The conceptual framework integrates all variables examined in the study into a single multilevel structure. County-level factors include access to grocery stores and supermarkets, fast-food restaurants and convenience stores, availability of farmers’ markets, including ones selling fruits and vegetables, recreation and fitness facilities, and sociodemographic conditions; these represent community-level exposures that shape daily opportunities for healthy behaviors. State-level factors include average SNAP benefits per capita and state soda taxes, and these highlight broader structural and policy environments that may mitigate or amplify local food landscape effects. Including both levels in a unified framework clarifies the theoretical basis for including these variables in a multilevel model and highlights how structural conditions at multiple levels may cumulatively shape adult obesity risk.

### 2.5. Variables and Measures

The dependent variable—adult obesity prevalence—was obtained from County Health Rankings (CHR) and represents the percentage of adults aged 18 years and older with a body mass index (BMI) ≥ 30. This measurement is based on self-reported height and weight from the Behavioral Risk Factor Surveillance System (BRFSS). To maintain temporal alignment among data sources, each CHR variable was matched with the closest available year in the USDA Food Environment Atlas. Food environment variables included several measures representing the availability and accessibility of retail food outlets. Grocery stores, convenience stores, supercenters, and fast-food restaurants were operationalized as county-level counts from the USDA Food Environment Atlas (2016), with the hypothesis that higher counts indicate greater exposure to each type of outlet. Farmers’ markets and farmers’ markets selling fruits and vegetables (2018) helped access use of locally grown produce that could be contributing to healthier food choices among diverse populations. SNAP-authorized stores (2017) provided the number of retailers participating in the Supplemental Nutrition Assistance Program. This variable served as a proxy for the accessibility of affordable and nutrition-supportive food outlets. Low-income, low-access households (2015) represented the percentage of residents living more than one mile (urban) or ten miles (rural) from a supermarket while also meeting the low-income criteria.

Physical activity environment characteristics were measured using county-level data on recreation and fitness facilities (2016), which reflected the density of commercial and public exercise facilities that may support active living. Prior research has shown that these facilities are associated with increased physical activity and lower obesity risk.

County-level socioeconomic factors included median household income (2015), poverty rate (2015), unemployment rate (2015), and food insecurity (2019). These variables highlight economic constraints that affect access to nutritious food and hence community health conditions. Demographic composition was represented using the percentage of population identifying as American Indian or Alaska Native (AIAN), Asian, Hispanic, Native Hawaiian or Pacific Islander (NHPI), Non-Hispanic Black (NHB), and Non-Hispanic White (NHW), taken from USDA Food Environment Atlas estimates. The racial/ethnic variables were included to examine racial disparities in obesity prevalence.

Finally, two state-level policy variables, soda tax (2014) and average SNAP benefits per capita (2024), were included to capture higher state-level policy contexts that may influence dietary behavior and food affordability. County-level variables were group-mean-centered and state-level variables were grand-mean-centered to distinguish within-state and between-state effects and to support interpretation in the multilevel model analysis.

Although the USDA Food Environment Atlas provides several measures in both count and density formats, we intentionally used count variables for food outlets and physical activity facilities for three reasons. First, the Atlas defines these indicators as structural characteristics of the county retail environment rather than population-normalized measures, and prior national studies using USDA data have similarly used raw counts to represent the absolute availability of food outlets [14,17,19]. Second, density measures (e.g., per 1000 residents or per square mile) can introduce substantial instability in sparsely populated or geographically large counties, where small changes in population size can disproportionately affect the calculated ratio. Given that many rural counties in the dataset have extremely low population density, using counts avoids the statistical distortions associated with denominators that vary widely across counties. Third, for indicators such as farmers’ markets and farmers’ markets selling fruits and vegetables, counts provide a more meaningful representation of availability because many counties have only one or two markets; converting these to density measures adds little interpretive value and can underestimate meaningful resource gaps. For these reasons, and consistent with published ecological analyses using CHR and USDA sources [7,14,19], count variables were retained in the final multilevel model.

### 2.6. Statistical Analysis

For this multilevel analysis, we applied a two-level random intercept linear model, with counties as level 1 units nested within their respective states at level 2. This structure accounts for the hierarchical nature of the data; counties in the same state often share policy settings, economic conditions, and other factors that could influence adult obesity prevalence. At the county level, the model included measures such as grocery store and farmers’ market counts, recreational facility access, income, poverty, unemployment, food insecurity, and racial/ethnic composition. At the state level, we added policy variables, including SNAP benefits per capita and food taxes. Allowing the intercepts to vary by state helped capture differences that the measured predictors could not fully explain. In this way, the model examined influences of both local and broader policy on obesity while still accounting for the fact that counties within a state are not entirely independent of one another.

To evaluate our study hypotheses, we applied this multilevel model by entering all county-level food, physical activity, and socioeconomic variables as fixed effects, followed by the state-level policy indicators. This allowed us to test whether the availability of food outlets, recreation facilities, and economic conditions were linked to adult obesity after accounting for broader state context. The model structure also made it possible to quantify how much of the variation in obesity occurs between states versus between counties. Taken together, this approach directly addressed our hypotheses by showing which local and state factors were most strongly associated with adult obesity when analyzed simultaneously.

#### Multilevel Model Specification

Level 1 (County-Level):

*Y_ij_* = *β*_0*j*_ + *β*_1_(% Low Access to Store, low income)*_ij_* + *β*_2_(Convenience Stores)_*ij*_ + *β*_3_(Grocery Stores)*_ij_* + *β*_4_(Farmers Markets)*_ij_* + *β*_5_(Farmers Markets Selling Fruits and Vegetables)*_ij_* + *β*_6_(Supercentres/Club Stores)_*ij*_ + *β*_7_(SNAP Authorized Stores)*_ij_* + *β*_8_(Fast-Food Restaurants)*_ij_* + *β*_9_(Recreation and Fitness Facilities)*_ij_* + *β*_10_(Population % Race)*_ij_* + *β*_11_(Median Household Income)*_ij_* + *β*_12_(Unemployment)*_ij_* + *β*_13_(Food Insecurity)*_ij_* + *β*_14_(Poverty Rate)*_ij_* + *ε_i_*, (1)

Level 2 (State-Level):

*β*_0*j*_ = *γ*_0_ + *γ*_1_(Median Household Income)*_j_* + *γ*_2_(SNAP Benefits)*_j_* + *γ*_3_(Soda Sales Tax)*_j_* + *γ*_4_(Chips/Pretzels Sales Tax)*_j_* + *u_j_* (2)

Combined Model:

*Y_ij_* = (*γ*_0_ + *γ*_1_(Median Household Income)*_j_* + *γ*_2_(SNAP Benefits)*_j_* + *γ*_3_(Soda Sales Tax)*_j_* + *γ*_4_(Chips/Pretzels Sales Tax)*_j_* + *u_j_*) + *β*_0*j*_ + *β*_1_(Low Access to Store)*_ij_* + *β*_2_(Convenience Stores)*_ij_* + *β*_3_(Grocery Stores)*_ij_* + *β*_4_(Farmers Markets)*_ij_* + *β*_5_(Farmers Markets Selling Fruits and Vegetables)*_ij_* + *β*_6_(Supercentres/Club Stores)*_ij_* + *β*_7_(SNAP Authorized Stores)*_ij_* + *β*_8_(Fast-Food Restaurants)*_ij_* + *β*_9_(Recreation and Fitness Facilities)*_ij_* + *β*_10_(Population % Race)*_ij_* + *β*_11_(Median Household Income)*_ij_* + *β*_12_(Unemployment)*_ij_* + *β*_13_(Food Insecurity)*_ij_* + *β*_14_(Poverty Rate)*_ij_* + *ε_ij_* (3)

This model enables the estimation of the association of county-level factors on adult obesity prevalence, considering the broader state context and accounting for variations at both the county and state levels. All analyses were conducted using STATA 19.

## 3. Results

### 3.1. Model Fit and Justification

The multilevel model was statistically justified with significant state-level variance (11.55, 95% CI: 7.61–17.53, *p* < 0.001) and county-level residual variance (4.78, 95% CI: 4.55–5.03). The likelihood ratio test confirmed multilevel modeling significantly improved fit over standard linear regression (χ^2^(1) = 2723.63, *p* < 0.001).

### 3.2. Descriptive Statistics of Key Variables

Table 1 presents overall descriptive statistics for adult obesity prevalence and the main county-level independent variables included in the multilevel model. These values summarize the distribution of food-environment, physical-activity, socioeconomic, and demographic indicators across all U.S. counties in the analytic dataset (N ≈ 3100). The table provides a baseline overview of county characteristics before examining multilevel associations.

### 3.3. State-Level Random Effects

**Figure 2:**
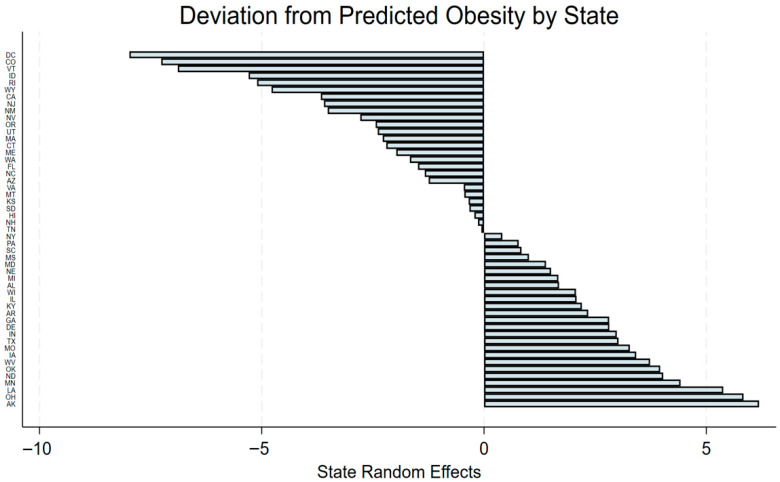
State-level random intercepts from the multilevel model.

Figure 2 displays the estimated state-level random intercepts from the multilevel model. These values represent how much each state’s average obesity prevalence deviates from the national expected level after accounting for all county-level predictors. Several states showed substantially higher or lower obesity levels than predicted, indicating that unmeasured contextual or policy factors likely contribute to geographic differences in adult obesity. This variation supports the decision to model states as a random intercept and illustrates the importance of accounting for shared state-level influences when evaluating county environments.

These deviations show that states differ systematically from the model’s expected obesity level, even after adjusting for county characteristics. Alaska (AK) had an obesity prevalence approximately 6.19 percentage points above the predicted value, followed by West Virginia (WV) (+3.74) and Texas (TX) (+3.02). In contrast, several states exhibited markedly lower-than-expected rates. Vermont (VT) showed a deviation of −6.89 percentage points, and Colorado (CO) was −7.26 below the model’s prediction. The District of Columbia (DC) displayed the largest negative deviation, at roughly −9.0 percentage points. These patterns suggest that unmeasured state-level conditions—such as regional policies, economic context, or cultural factors—may influence obesity risk beyond county-level environments.

### 3.4. Fixed Effects Results

Table 2 summarizes the significant fixed-effects results from the multilevel model. The table highlights only predictors that reached statistical significance (*p* < 0.05), including food environment indicators, socioeconomic factors, and racial/ethnic composition variables

#### Multilevel Model Results Key Findings

The multilevel model included 14 predictors spanning food environment characteristics, physical activity access, socioeconomic conditions, and racial/ethnic composition. Several county-level and state-level variables showed significant associations with adult obesity prevalence.

Greater densities of fast-food restaurants (+0.061), convenience stores (+0.089), and higher proportions of low-access store areas (+0.169) were each associated with higher adult obesity rates. In contrast, a greater number of supercenters (−0.053) and higher access to recreational facilities (−0.028) were linked to lower obesity prevalence. Grocery stores and farmers’ markets, including those selling fruits and vegetables, were not significant predictors in the final model.

At the state level, higher Supplemental Nutrition Assistance Program (SNAP) benefits per capita (−0.058) were associated with lower obesity rates, while state food taxes were not statistically significant.

County racial and ethnic composition also showed meaningful associations. Higher proportions of Asian (−0.158) and non-Hispanic White residents (−0.020) were associated with lower obesity prevalence. Higher proportions of American Indian/Alaska Native (+0.185), Hispanic (+0.121), and non-Hispanic Black residents (+0.025) were associated with higher prevalence.

Among socioeconomic variables, higher median household income (−0.0004) corresponded with lower obesity rates, while higher levels of poverty (+0.307), food insecurity (+0.282), and unemployment (+0.196) were associated with higher rates.

### 3.5. State-Level Heterogeneity in Key Predictors

To further explore geographic variation, coefficient plots were used to visualize how the effect of selected predictors—such as food insecurity and recreational access—varies across states. These exploratory plots are based on separate OLS models stratified by state.

Across most states, the coefficient plot shows a positive association between food insecurity and adult obesity prevalence. Many of the confidence intervals cross zero also effect sizes differ across state, indicating that not all associations are statistically significant. These differences highlight the need for localized strategies that address both food insecurity and the availability of healthy food options.

All coefficient plots for other predictors (e.g., recreational access, racial composition) are available in Appendix B. A Summary table with multi-level model results (Appendix A) and a summary (Appendix B, Table A2) synthesizing all state-stratified OLS model results is also provided.

## 4. Discussion

Because this is a cross-sectional ecological study, the associations reported here cannot be interpreted as causal. The results reflect patterns of associations across U.S. counties and should be viewed as hypothesis-generating rather than demonstrating direct effects. The multilevel analysis revealed several consistent associations: counties with higher food insecurity, poverty, unemployment, and a greater proportion of low-access households tended to have higher adult obesity prevalence, whereas counties with more recreational facilities and higher median household income tended to have lower prevalence [5,11,16,20,21]. Fast-food and convenience-store density showed positive associations with adult obesity prevalence, while grocery stores and farmers’ markets were not significantly associated in the final model. At the state level, higher Supplemental Nutrition Assistance Program (SNAP) benefits per capita showed a modest protective association, whereas state food taxes were not significant, despite prior arguments that fiscal measures such as sugar-sweetened beverage taxes may improve population health outcomes [22]. These findings are consistent with previous studies showing that structural disadvantage, limited access to health-promoting environments, and economic constraints are consistently correlated with higher obesity prevalence across U.S. communities [23].

Although grocery stores and farmers’ markets are often assumed to be protective, prior research has reported inconsistent associations, with several national studies finding that access alone does not guarantee healthier diets or lower obesity rates if affordability, food quality, and cultural relevance are not also addressed [12,17,20,24,25,26]. The present findings support this emerging evidence by suggesting that availability may not be sufficient unless accompanied by economic capacity and high-quality, culturally appropriate food options.

In contrast, the positive association between food insecurity and adult obesity is consistent with the well-documented “food insecurity–obesity paradox,” where constrained food budgets can lead households to rely on calorie-dense, low-cost foods [11,15,16]. Again, greater availability of recreational facilities was seen to be associated with lower adult obesity prevalence rates in this analysis. These findings align with prior work showing that access to parks, trails, and fitness centers can support physical activity and healthier lifestyles [7,8,9,13].

Taken together, these findings provide a more nuanced picture of how multiple community conditions, economic, environmental, and policy-related, may jointly shape obesity patterns across counties.

Because of the ecological and cross-sectional design, these findings cannot be used to make causal claims or to recommend specific policy actions. Rather, they may help identify community characteristics that appear repeatedly associated with obesity across diverse contexts and that may warrant further investigation. Any policy-related statements should therefore be interpreted as potential avenues for exploration, not as prescriptive recommendations.

The patterns observed in this study are broadly consistent with prior national and regional research on community determinants of obesity. Earlier studies have shown that food insecurity, poverty, and limited availability of recreational resources are frequently linked with higher obesity prevalence across U.S. communities, particularly in economically disadvantaged or rural areas, and the present findings echo these trends [5,14,16,23]. Like previous ecological analyses, fast-food and convenience-store density were positively associated with adult obesity, whereas grocery store and farmers’ market availability showed weaker or inconsistent associations—an outcome that several earlier studies have also noted [6,10,12]. The modest inverse association between SNAP benefit levels and obesity aligns with work suggesting that greater economic support may help buffer food insecurity-related risk, although evidence remains mixed across studies [15,16,26]. Again, some results differ from earlier findings; for example, grocery store and farmers’ market counts were not significant predictors in the final model, whereas prior studies have reported protective associations in certain settings. These differences may reflect geographic heterogeneity, measurement variation, or contextual factors that were captured at the county level. Collectively, the study contributes to updated national-level evidence that reinforces several established relationships while also highlighting areas, particularly the limited impact of some food-access measures—where additional inquiry is warranted.

## 5. Summary and Conclusions

This study examined how characteristics of the food environment, physical-activity environment, socioeconomic context, and state-level policy conditions are correlated with adult obesity across U.S. counties. Higher obesity prevalence tended to appear in counties characterized by food insecurity, poverty, unemployment, and fewer recreational facilities, while somewhat lower rates appeared in counties with higher income and more access to places for physical activity. State-level SNAP benefit levels were modestly associated with lower obesity prevalence, while grocery stores, farmers’ markets, and food taxes showed no consistent associations. These results align with prior research emphasizing structural disadvantage as a key factor associated with obesity and contribute additional evidence from a multilevel, nation-wide perspective. Because these findings are correlational, they should be viewed as indicative of patterns that may be examined in more depth through longitudinal or natural experiment designs.

## 6. Scope and Limitations

This study has several important limitations. There is a temporal mismatch in the data, as variables were drawn from different years (2014–2024) based on availability. However, because food environment variables typically change slowly over time, this limitation is unlikely to substantially affect the analysis. Measurement limitations exist, wherein there is use of raw counts that do not capture outlet size, quality, or food healthfulness, again the “Low Access to Store” variable does not account for public transportation or informal access methods. Ecological design of the study and the cross-sectional nature precludes causal inference and raises potential for reverse causality bias. there are omitted variables like zoning laws, physical activity initiatives, cultural food preferences, and individual-level factors, that were excluded due to data unavailability. Because County Health Rankings derives obesity prevalence from BRFSS self-reported height and weight data, with model-based estimation for small counties, some residual bias may remain despite these adjustments.

## 7. Recommendations for Future Research

Future studies should utilize census tract/ZIP code level datasets for better spatial heterogeneity, expand cross-level interactions to isolate race/ethnicity effects, include sociocultural variables addressing acculturation and food practices, employ natural experiments to study policy changes over time, and adopt mixed-methods approaches combining quantitative analysis with qualitative community perspectives to guide more responsive and equitable policy solutions.

## Figures and Tables

**Table 1:** Descriptive characteristics of key study variables across U.S. counties (N ≈ 3142). Values shown are mean (SD) and range (min–max) for the primary outcome (adult obesity) and key food environment, socioeconomic, and physical activity indicators included in the multilevel model.

Variable	Mean (SD)	Min–Max	Description
Adult obesity (%)	32.1 (4.8)	18.0–48.0	County-level adult obesity prevalence, CHR 2022 [18]
Fast-food restaurant count	63.4 (82.7)	0–1120	Number of limited-service restaurants, FEA 2016 [19]
Grocery stores (count)	14.8 (21.6)	0–285	Number of supermarkets and grocery stores, FEA 2016 [19]
Convenience stores (count)	44.2 (53.0)	0–610	County-level convenience stores, FEA 2016 [19]
Supercenters (count)	3.1 (3.9)	0–42	Walmart/large superstores, FEA 2016 [19]
Recreational facilities (count)	9.5 (16.2)	0–230	Fitness/recreational facilities, CHR/FEA 2016 [18,19]
Food insecurity (%)	13.1 (3.4)	6.0–30.0	% of population lacking reliable access to food, CHR 2018 [18]
Median Household Income (USD)	52,300 (12,400)	31,000–105,000	Median county income, CHR 2015 [18]
Poverty (%)	15.2 (6.4)	3.0–45.0	County population below poverty line, CHR 2015 [18]
% Hispanic	10.2 (14.6)	0–98	Racial/ethnic composition, FEA 2021 [19]
% Non-Hispanic Black	9.1 (14.9)	0–87	Racial/ethnic composition, FEA 2021 [19]
% Non-Hispanic White	74.4 (20.7)	2–99	Racial/ethnic composition, FEA 2021 [19]

**Table 2:** Summary of significant predictors of adult obesity in the multilevel model.

Predictor	Direction of Association	Coefficient (β)	Significance
Fast-food restaurant density	↑ Higher obesity	+0.061	*p* < 0.05
Convenience store density	↑ Higher obesity	+0.089	*p* < 0.05
Low-access and low-income %	↑ Higher obesity	+0.169	*p* < 0.05
Supercenter density	↓ Lower obesity	−0.053	*p* < 0.05
Recreational facility access	↓ Lower obesity	−0.028	*p* < 0.05
SNAP benefits per capita (state-level)	↓ Lower obesity	−0.058	*p* < 0.05
% AIAN	↑ Higher obesity	+0.185	*p* < 0.05
% Hispanic	↑ Higher obesity	+0.121	*p* < 0.05
% NHB	↑ Higher obesity	+0.025	*p* < 0.05
% Asian	↓ Lower obesity	−0.158	*p* < 0.05
% NHW	↓ Lower obesity	−0.020	*p* < 0.05

Note: ↑ indicates a positive association with adult obesity prevalence (higher values of the predictor are associated with higher obesity). ↓ indicates a negative association with adult obesity prevalence (higher values of the predictor are associated with lower obesity). Coefficients (β) are from multilevel linear regression models; statistical significance is defined as *p* < 0.05.

**Figure A1:**
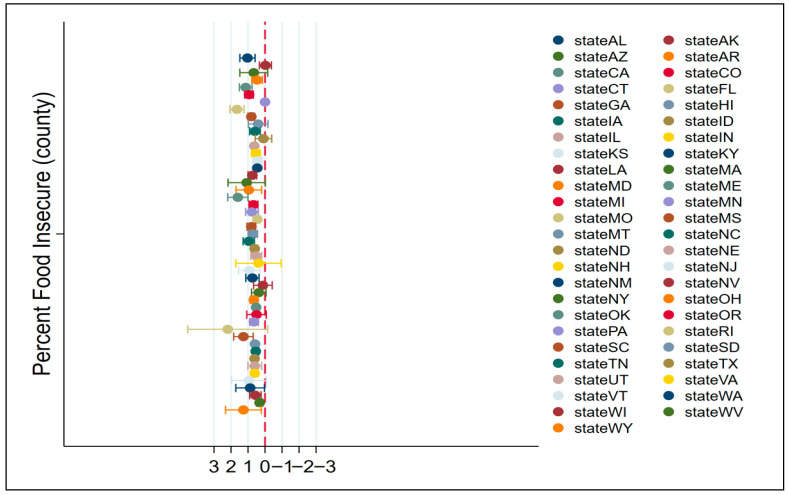
Effect of percent food insecure on adult obesity by state (state-stratified OLS models). Each point indicates the estimated regression coefficient for an individual state; horizontal bars reflect 95% confidence intervals; the vertical dashed line represents no association (0).

**Figure A2:**
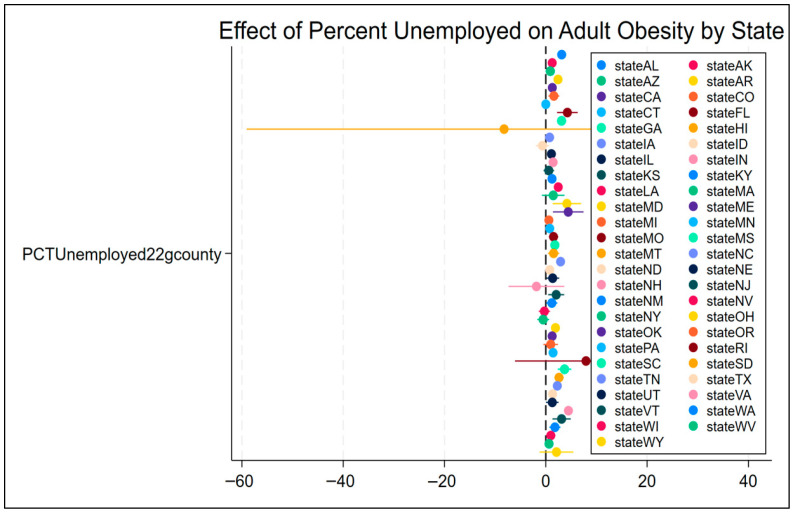
Coefficient plot—effect of county-level unemployment on adult obesity rates by state.

**Figure A3:**
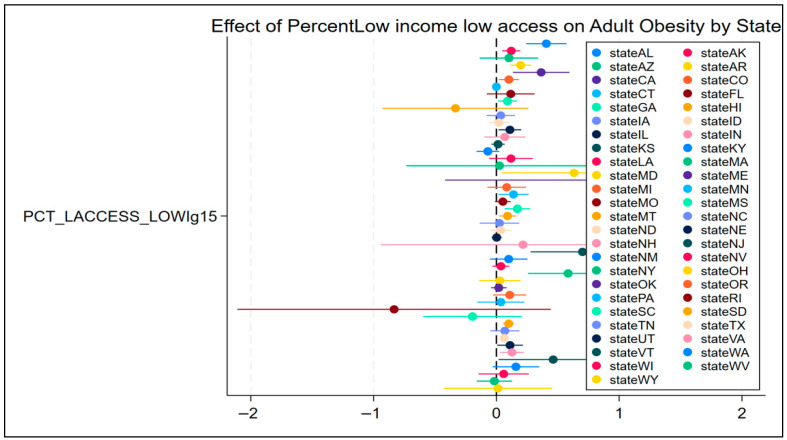
Coefficient plot—association of low access and low income with obesity prevalence by state.

**Figure A4:**
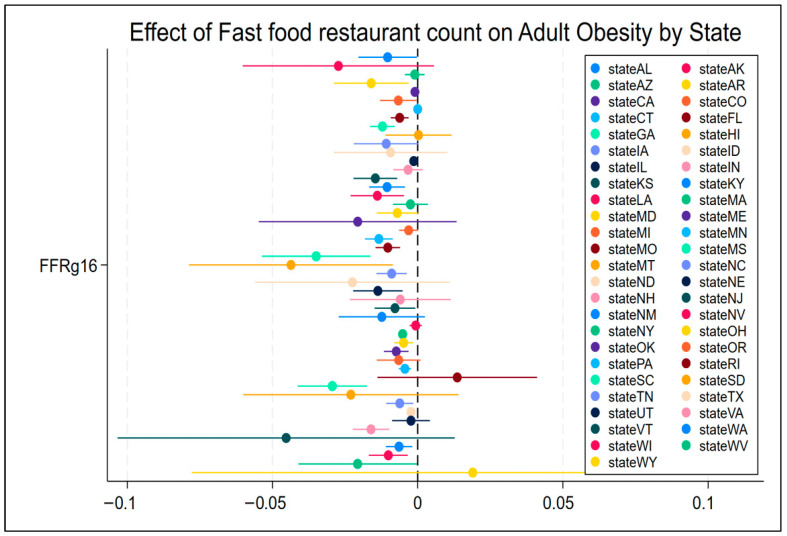
Effect of fast-food count on adult obesity rates by state.

**Figure A5:**
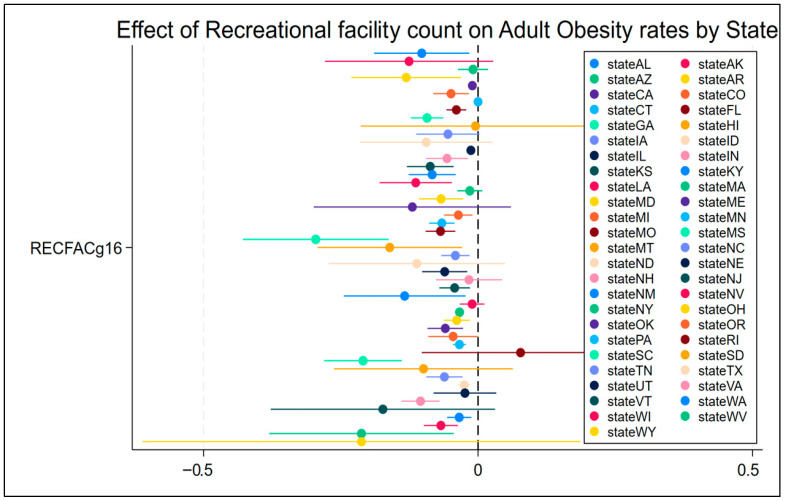
Coefficient plot: association of recreation facilities (Recfac16) and adult obesity prevalence by state.

**Figure A6:**
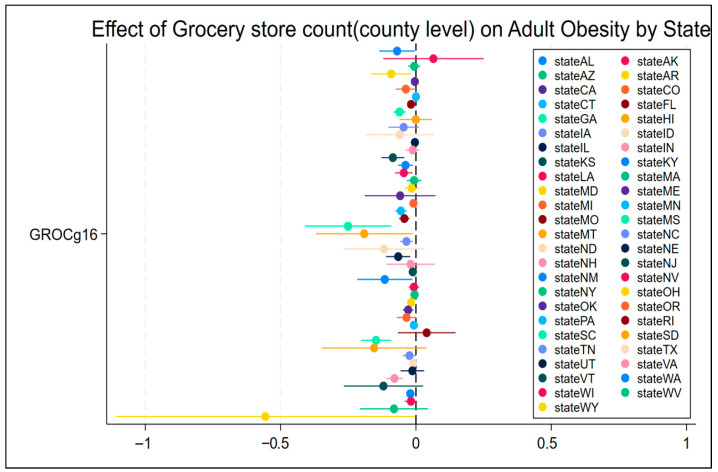
Coefficient plot: association of grocery stores (Grocg16) and adult obesity prevalence by state.

**Figure A7:**
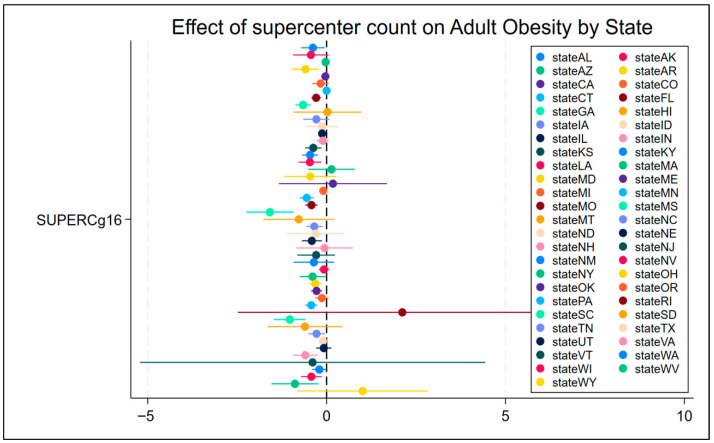
Coefficient plot—association of supercenters (Supercg16) and obesity prevalence by state.

**Figure A8:**
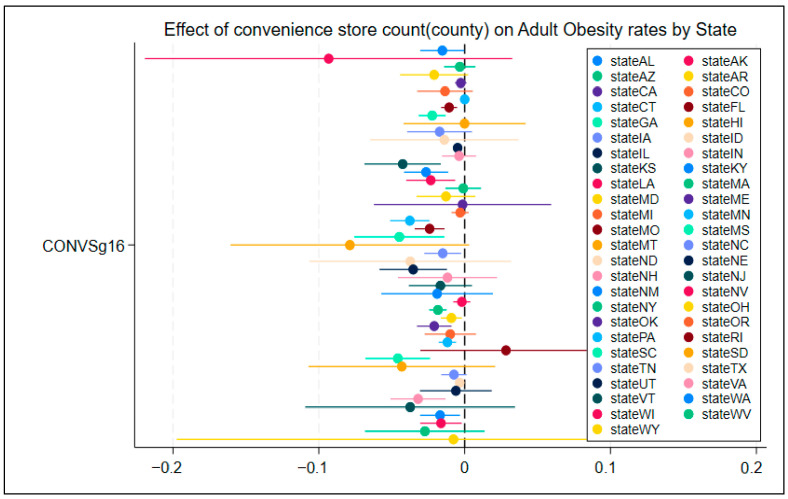
Coefficient plot—association between convenience store count (convsg16) and obesity prevalence by state.

**Figure A9:**
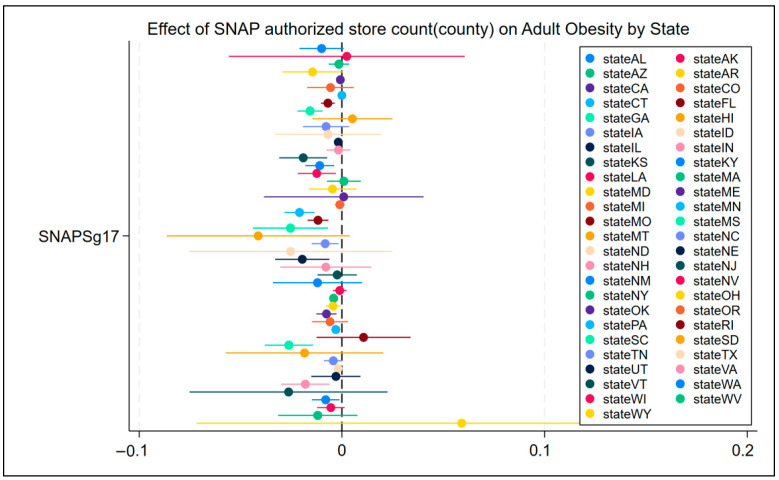
Coefficient plot-association between SNAP-authorized store count and adult obesity prevalence by state.

**Figure A10:**
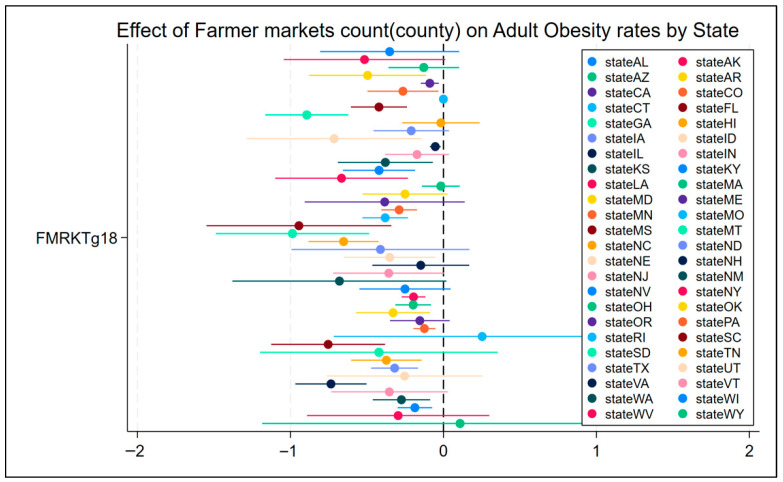
Coefficient plot—association between farmers’ markets and adult obesity.

**Figure A11:**
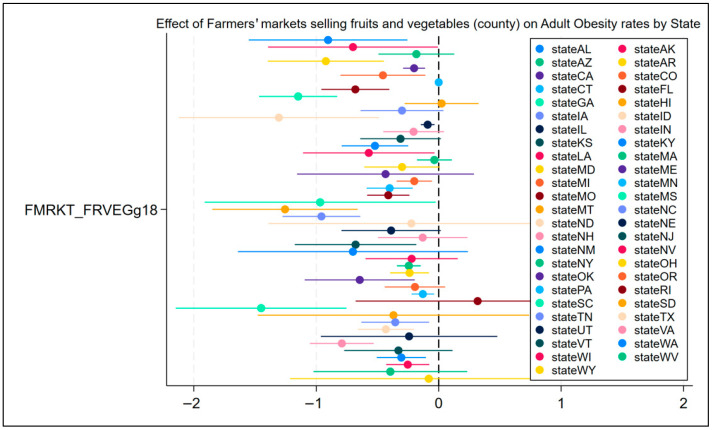
Coefficient plot—association between farmers’ markets selling fruit and vegetables and adult obesity rates.

**Figure A12:**
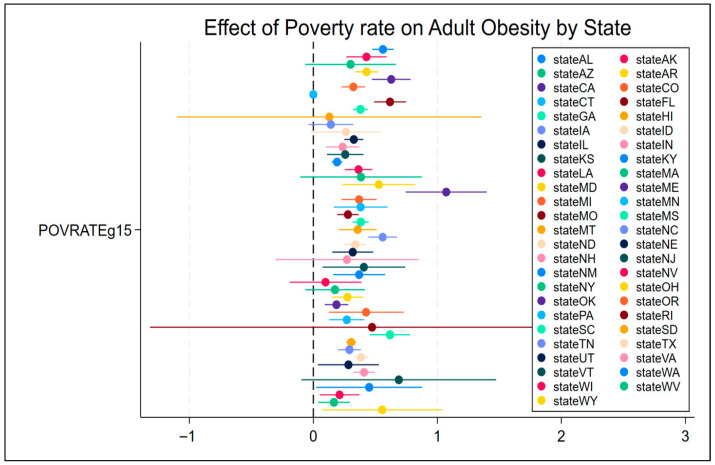
Coefficient plot—association between county-level poverty rate on adult obesity.

**Figure A13:**
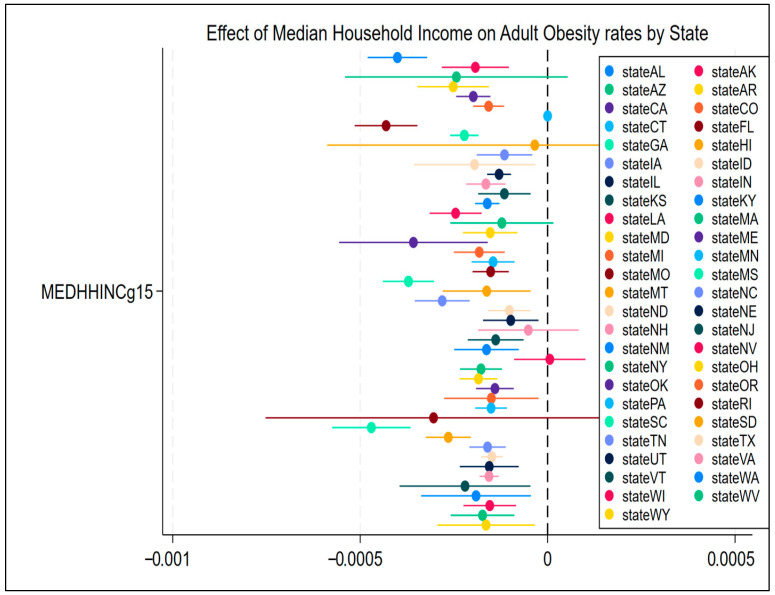
Coefficient plot—association between county-level median household income rate on adult obesity rates by state.

**Figure A14:**
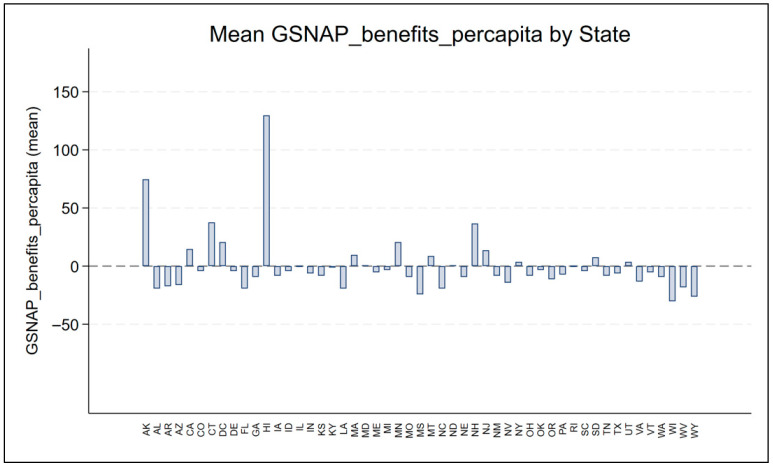
SNAP benefits per capita (GSNAP_benefits_percapita): policy-leveraged (grand-mean-centered values).

## Data Availability

No new data were created or analyzed in this study. The data analyzed are publicly available from sources cited in the manuscript.

## Abbreviations

The following abbreviations are used in this manuscript:

CDC	Center of Disease Control and Prevention
SNAP	Supplemental Nutrition Assistance Program.
USDA	United States Department of Agriculture
PLACES	Population Level Analysis and Community Estimates.
BMI	Body Mass Index

## Appendix A

**Table A1:** MLM Summary Table.

Variable	Coeff.	Std. Error	*p*-Value	Interpretation
Grocery store (GROCg16)	−0.01	0.014	0.46	Not statistically significant; no clear evidence grocery store density affects obesity rates.
Farmers’ market (FMRKTg18)	−0.006	0.0096	0.55	Not statistically significant; no significant association with obesity.
Farmers’ market selling fruits and vegetables (FMRKT_FRVEGg18)	−0.0	0.009	0.13	Not statistically significant; no significant effect.
Fast-food Restaurants. (FFRg16)	0.0612	0.014	0.00	A 1-unit increase in fast-food density (FFRg16) is associated with a 0.0612-percentage-point increase in adult obesity rates. This is a significant and positive association; more fast-food restaurants are linked to higher obesity rates.
Supercenter (SUPERCg16)	−0.053	0.019	0.01	A 1-unit increase in supercenter density (SUPERCg16) is associated with a 0.053-percentage-point decrease in adult obesity rates. This is a significant and negative association; more supercenters are linked to lower obesity, contrary to the hypothesis.
Convenience store (CONVSg16)	0.0885	0.019	0.001	A 1-unit increase in convenience store density (CONVSg16) is associated with a 0.0885-percentage-point increase in adult obesity rates. This is a significant and positive association; more convenience stores are associated with higher obesity.
% low access to stores, low income (PCT_LACCESS_LOWIg15)	0.169	0.034	0.001	A 1-percentage-point increase in low-income households with low access to stores (PCT_LACCESS_LOWIg15) is associated with a 0.1686-percentage-point increase in adult obesity rates. This significant positive association indicates that limited access to food outlets is linked to higher obesity prevalence.
(RECFACg16) recreational facilities	−0.028	0.006	0.001	A 1-unit increase in recreational facility density (RECFACg16) is associated with a 0.0276-percentage-point decrease in adult obesity rates. This is a significant and negative association; more recreational facilities are linked to lower obesity.
SNAP-authorized stores (SNAPSg17)	0.002	0.001	0.09	The result is not statistically significant.
SNAP benefits per capita (GSNAP_benefits_percapita)	−0.058	0.01	0.001	A 1-unit increase in GSNAP_benefits_percapita is associated with a 0.0575-percentage-point change in adult obesity rates. This is a significant and negative association; higher SNAP benefits are per capita linked to lower obesity.
Soda tax stores (GSODATAX_STORES14)	−0.378	0.293	0.21	Not statistically significant.
Chips/pretzel tax stores (GCHIPSTAX_STORES14)	−0.712	0.474	0.13	Not statistically significant.
Soda tax vending machine (GSODATAX_VENDM14)	0.207	0.653	0.76	Not statistically significant.
Chips/pretzel tax vending machine (GCHIPSTAX_VENDM14)	−0.527	0.396	0.19	Not statistically significant.
% food insecurity (PCTFoodInsecure21gCounty)	0.282	0.009	0.001	Statistically significant. A 1-percentage-point increase in county-level food insecurity is associated with a 0.28-percentage-point increase in adult obesity, supporting the hypothesis that greater food insecurity contributes to higher obesity rates.
% unemployed (PCTUnemployed22gcounty)	0.196	0.02	0.001	A 1-percentage-point increase in county-level unemployment is associated with a 0.1958-percentage-point increase in adult obesity rates. This strong positive association supports the hypothesis that economic insecurity contributes to obesity risk.
Race/Ethnicity Controls				
% Asian (PCT_ASg23)	−0.158	0.02	0.00	A 1-percentage-point increase in Asian population (PCT_AIANG21g) is associated with a 0.158-percentage-point decrease in adult obesity rates. This is a significant and negative association; a higher Asian population is linked to decreased obesity.
% American Indian/Alaskan Native (PCT_AIANg23)	0.185	0.022	0.001	A 1-percentage-point increase in American Indian/Alaskan Native population (PCT_AIANG21g) is associated with a 0.1848-percentage-point increase in adult obesity rates. This is a significant and positive association; a higher American Indian/Alaskan Native population is linked to increased obesity.
% Native Hawaiian Pacific Islander (PCT_NHPIg23)	−0.023	0.057	0.69	Not statistically significant.
% Hispanic (PCT_H23g)	0.121	0.015	0.001	A 1-percentage-point increase in Hispanic population (PCT_Hisp21g) is associated with a 0.121-percentage-point increase in adult obesity rates. This is a significant and positive association; a higher Hispanic population is linked to increased obesity.
% Non-Hispanic Black (PCT_NHBg23)	0.025	0.01	0.02	A 1-percentage-point increase in Non-Hispanic Black population (PCT_NHBg23) is associated with a 0.025-percentage-point increase in adult obesity rates. This is a significant and positive association; more Non-Hispanic Black residents are linked to higher obesity.
% Non-Hispanic White (PCT_NHWg23)	−0.02	0.009	0.03	A 1-percentage point-increase in Non-Hispanic White population (PCT_NHWg23) is associated with a 0.02-percentage-point decrease in adult obesity rates. This is a significant and negative association; more Non-Hispanic White residents are linked to lower obesity.
Median household income (MEDHHINCg15)	−0.0001	0.0001	0.001	A 1-unit increase in median household income (MEDHHINCg15) is associated with a 0.0004-percentage-point decrease in adult obesity rates. This is a significant and negative association; higher income is linked to lower obesity.
Poverty rate (POVRATEg15)	0.307	0.047	0.00	A 1-percentage-point increase in poverty rate (POVRATEg15) is associated with a 0.3065-percentage-point increase in adult obesity rates. This is a significant and positive association; higher poverty is associated with higher obesity.

## Appendix B

State-level effects—food insecurity with adult obesity rates.

State level effects of percent unemployed on adult obesity rates.

State-level effects—association of low access and low income with adult obesity rates.

Fast-food restaurant density (FFR16): clear policy relevance.

State-level effects—recreation facility count and adult obesity.

State-level effects—grocery store count and adult obesity.

State-level effects—supercenter count and adult obesity.

State-level effects—convenience stores and adult obesity.

State-level effects—SNAP-authorized stores and adult obesity.

State-level effect of farmers’ markets on adult obesity.

State-level effect of farmers’ markets selling fruits and vegetables on adult obesity.

State-level effect of county-level poverty rate on adult obesity.

State-level effect of county-level median household income on adult obesity.

Additional predictor visualizations and notes.

The bar graph displays the mean SNAP benefits per capita (GSNAP_benefits_percapita) across U.S. states. A few states, such as DC and Alaska, provide higher SNAP benefits per capita, while several others fall below the mean. Other states show negative-centered values due to grand-mean centering. In multilevel models, this variable demonstrated perfect multicollinearity with the state random intercepts, leading STATA to automatically omit it. Because of this, regression analysis was not feasible, and instead, visualizing the distribution through a bar graph provided a meaningful way to highlight inter-state differences in SNAP support.

**Table A2:** Summarizes coefficient plots for all variables.

Variable	General Trend	Key Finding
Fast-Food Restaurant Density (FFRg16)	Combination of negative and positive, some significant	Fast-food density linked to higher obesity in some states but not in others
Percent Unemployed (PCTUnemployed22gcounty)	Mixed, several negative	Unemployment negatively associated with obesity in some states
Percent Food Insecure (PCTFoodInsecure21gCounty)	Mixed, mostly non-significant	No consistent pattern: some states show weak positive link
Percent Low Access to stores + Low Income (PCT_LACCESS_LOWIg15)	Mixed, weak effects	Low income, low access not consistently associated
Grocery Store (GROCg16)	Mostly negative, some significant	Greater grocery access linked to lower obesity. In many states, more grocery stores are linked to lower obesity rates, supporting access to healthy food
Supercenter (SUPERCg16)	Highly variable, wide CI	Unstable estimates; some positive associations. Some states show a link to higher obesity
Convenience Store (CONVSg16)	Mixed, mostly non-significant	Inconclusive; substitution effects possible (a substitute to grocery stores with varying impact on dietary quality either positively or negatively, adding complexity to the relationship)
SNAP-Authorized Stores (SNAPSg17)	Weak, mixed	SNAP store density not a strong predictor
Recreational Facilities (RECFACg16)	Mostly negative, some significant	Access to recreation linked to lower obesity in many states
Farmers’ Markets (FMRKTg18)	Mixed, wide CI	Mixed influence; variability across states
Farmers’ Markets Selling Fruits and Vegetables (FMRKT_FRVEGg18)	Mostly negative, some significant	States with more markets selling fruits/vegetables show reduced obesity in some regions
Percent American Indian/Alaska Native (PCT_AIANg23)	Wide CI, mostly non-significant	Estimates unstable due to small subgroup size
Percent Asian Population (PCT_ASg23)	Mostly negative, some significant	A larger Asian population is often linked to lower obesity rates, consistent with national trends
Percent Hispanic Population (PCT_Hg23)	Mixed, few negative	Some states show lower obesity with higher Hispanic population, though effects vary
Percent Native Hawaiian/Pacific Islander (PCT_NHPIg23)	Wide CI, outliers present	High variability due to small populations
Percent Non-Hispanic Black (PCT_NHBg23)	Mixed, slightly positive	Some positive associations observed: some states show increased obesity with higher Black population; not uniform
Percent Non-Hispanic White (PCT_NHWg23)	Mostly negative.	Generally protective: higher White population linked to lower obesity
Median Household Income (MEDHHINCg15)	Strongly negative in most states	Consistently associated with lower obesity prevalence; higher income is a protective factor
Poverty Rate (POVRATEg15)	Mixed, some positive	Poverty shows variable influence; some states show higher obesity with higher poverty
